# Epidemiology and zoonotic potential of Livestock-associated *Staphylococcus aureus* isolated at Tamil Nadu, India

**DOI:** 10.1186/s12866-023-03024-3

**Published:** 2023-11-04

**Authors:** Relangi Tulasi Rao, Vinoth Madhavan, Pavitra Kumar, Gnanaraj Muniraj, Natesan Sivakumar, Jayakumar Kannan

**Affiliations:** 1 Department of Animal Behaviour & Physiology, School of Biological Sciences, Madurai Kamaraj University, Tamil Nadu, Madurai, 625021 India; 2 Vascular Biology Laboratory, AU-KBC Research Centre, Anna University, Tamil Nadu, Chennai, 600044 India; 3 Department of Biotechnology, Bishop Heber College, Tamil Nadu, Tiruchirapalli, 620017 India; 4 Department of Molecular Microbiology, School of Biotechnology, Madurai Kamaraj University, Tamil Nadu, Madurai, 625021 India

**Keywords:** Antibiogram, Epidemiology, Livestock, *Staphylococcus aureus*, Enterotoxins, Zoonosis

## Abstract

**Background:**

*Staphylococcus aureus* is part of normal flora and also an opportunistic pathogen responsible for a wide range of infections in both humans and animals. Livestock-associated *S. aureus* (LA*-SA*) has gained importance in recent years due to its increased prevalence in recent years, becoming a worry in public health view. This study aimed to study the epidemiology of LA-SA strains in Madurai district, Tamil Nadu, India.

**Methods:**

A total of 255 samples were collected from bovine and other small ruminants like goats and sheep nares (*n* = 129 and *n* = 126 respectively). Nasal swab samples were collected from study animals with sterile sample collecting cotton swabs (Hi-Media, Mumbai). Samples were transported to the lab in Cary-Blair Transport media for further analysis. The samples were tested for *S. aureus* using antibiotic selection and PCR-based assays. The pathogenicity of the bacteria was assessed using chicken embryo models and liver cross-sections were used for histopathology studies.

**Results:**

The prevalence rate in bovine-associated samples was 42.63% but relatively low in the case of small ruminants associated samples with 28.57% only. The overall prevalence of *S. aureus* is found to 35.6% and MRSA 10.98% among the study samples. The antibiogram results that LA*-SA* isolates were susceptible to aminoglycosides and tetracyclines but resistant to β-lactam drugs. The biofilm formation results showed that the LA-*SA* isolates are weak to high-capacity biofilm formers. The enterotoxigenic patterns revealed that most of the isolated strains are enterotoxigenic and possess classical enterotoxins. The survival analysis of chicken embryos suggested that the Bovine-associated strains were moderately pathogenic.

**Conclusion:**

The study concluded that economically important livestock animals can act as reservoirs for multi-drug resistant and pathogenic which in-turn is a concern for public health as well as livestock health.

**Supplementary Information:**

The online version contains supplementary material available at 10.1186/s12866-023-03024-3.

## Introduction

*Staphylococcus aureus* is a Gram-positive bacterium and considered as an opportunistic pathogen that is capable of colonizing different animal species [1]. *S. aureus* could able to cause a set of infections in economically important livestock animals including cows, sheep, goats, poultry and rabbits. In veterinary etiology, *S. aureus* is cited as the causative agent of mastitis, which is a mammary gland infection in dairy cows, small ruminants [2, 3]. It is also a major cause of lameness in the poultry industry, the result of skeletal infections in commercial broiler chickens [4], as well as being identified as a pathogen of farmed rabbits [5].

The discovery of antibacterial drugs in the mid-twentieth century revolutionized the treatment strategies of bacterial infections. But later antibiotic resistance became a primary concern in treating bacterial diseases. Antibacterial resistance is considered as the ultimate One Health issue [6]. The pattern of antimicrobial susceptibility varies according to the degree and duration of livestock exposure to antimicrobials [7]. Since antimicrobials are commonly used in livestock as growth promoters, such exposure is threatening because it facilitates the evolution of antimicrobial resistance in the associated bacteria. Over the last few decades, animal-associated *S. aureus* strains have globally emerged as multidrug-resistant strains among livestock animals, as well as among people who have contact with food-producing animals [8].

In this modern era, the emergence of new zoonotic diseases and the re-emergence of zoonotic diseases has become a major concern among veterinarians. The importance of zoonoses in the rise of human infections cannot be exaggerated. Around 61% of human microbial pathogens and 73% of emerging human pathogens identified during the past two decades are zoonotic. The emergence of multi-drug resistant *S. aureus* (MDR-*SA*) became a global menace in the new millennium [9]. Besides its zoonosis, *S. aureus* is dangerous because of its harmful effects on animal health, which in turn has a huge impact on the welfare of animals and causes major economic losses in livestock production.

The epidemiological knowledge of LA*-SA* in Tamil Nadu, India is also scanty. Furthermore, the emergence of LA-MRSA is on the rise and a big worry in terms of public health. Human-animal interactions, especially between humans and livestock-animals are quite common due to the domestication of animals, especially in India. Estimating the pathogenic potentiality of the livestock-associated *S. aureus* strains is necessary for understanding if it is a zoonotic agent or are the livestock animals responsible and acting as reservoirs. The study, primarily, is focused on the epidemiological estimation of LA-*SA* strains, and intra- and inter-strain comparison of the variations in virulence capacity in LA-*SA* strains isolated from Madurai district, Tamil Nadu, with the aid of in vivo studies.

## Materials and methods

### Sample collection

**Fig. 1:**
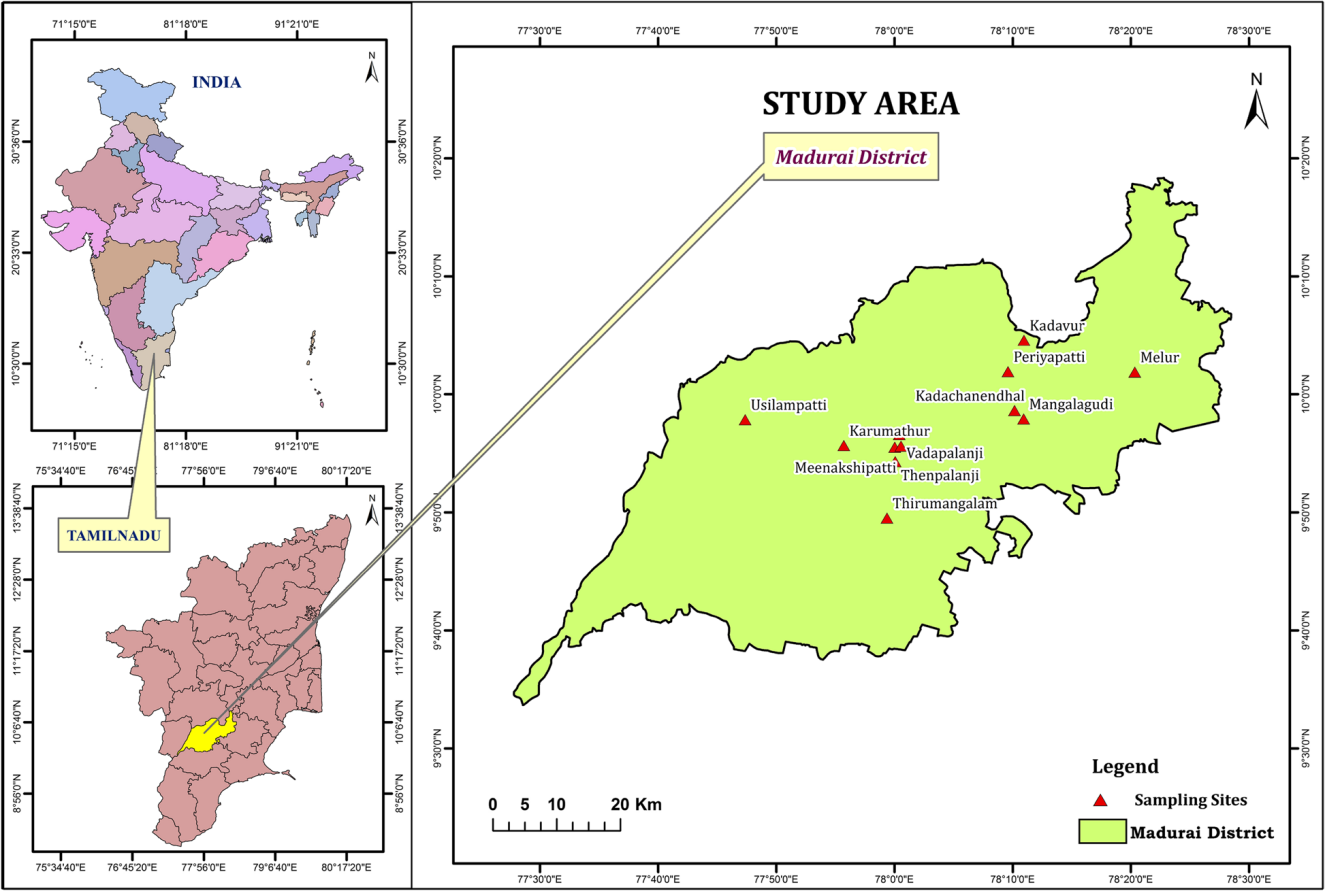
Sampling points—Map of location and sampling points of the study area in Madurai district, Tamil Nadu, India

The random sampling method was followed to collect the samples. Nasal swabs collected from adult healthy animals including cows (Bovines) and small ruminants (Caprine and Ovine) with sterile cotton swabs (Hi-Media Sterile culture collecting device) in Madurai district and sampling points were denoted in the location map (Fig. 1). Collected samples were transported to the laboratory in Cary-Blair transport media. The samples were processed on the day of sample collection itself to ensure the maximum recovery of bacteria [10]. Samples, not more than five, were collected to ensure heterogeneity from any herd or farm.

### Plate-based identification of *S. aureus*

The nasal swabs collected initially spread on Mannitol Salt Agar (MSA) and Nutrient Agar with 7.5% of NaCl, a selective medium for *S. aureus*. Plates were incubated at 37 °C for 24 – 48 h. Further, Gram staining was performed with a conventional established protocol for positive isolates on Agar plates. For Coagulase test, freshly prepared 12 to 18 h broth cultures were inoculated over the Baird-Parker agar plate with the addition of Egg yolk tellurite emulsion (Hi-Media, India) and plates were incubated at 37 °C for 24 – 48 h. After incubation, the positive strains form black or brownish-black color colonies which indicate the presence of Coagulase positive strains.

### Identification of *S. aureus* by multiplex PCR

DNA isolation performed with the aid of the Favorogen Tissue DNA extraction kit, Taiwan. DNA extracted according to the Manufacturer’s protocol. Lysostaphin (Sigma, USA) used for better lysis of cell walls as per manufacturer's recommendation. Quantification analysis was performed on Nanodrop 2000 (Thermo Scientific, USA). Multiplex PCR reactions have been carried out based on standard methods to characterize *S. aureus* at the species level and methicillin resistance [11–13]. Multiplex PCR reactions carried out with the *Staphylococcus*-specific *16 s rRNA*, *femA* and *tetM* primers to confirm the strains.

### Antibiogram

Antibiotic susceptibility tests performed on all *S. aureus* isolates to determine their antibiotic-sensitivity pattern according to Kirby-Bauer [14] protocol. Multiple Drug Resistant (MDR) phenotypes recorded for isolates showing resistance to four and more antibiotics [15]. The antibiogram results were interpreted according to CLSI guidelines. Test strains were screened for the β-Lactamase production for the further confirmation of resistance towards β-lactum drugs [16] [(Protocols mentioned in Supplementary file 1)].

### Assessment of biofilm formation

In brief, the *S. aureus* strains were grown in LB broth for overnight at 37 °C, then the overnight grown cultures were diluted to 1:100 into fresh medium and 100 µl of dilution was transferred to 96-well microtitre plate. For the quantitative assay, four replicate wells kept for treatment. Microtitre plates were incubated for overnight at 37 °C. For staining biofilm grown in microtitre plates, and biofilm quantification was carried out according to the protocol [17] (detailed protocol was given in Supplementary File 1) by O’Toole (2011).

### In vivo estimation of virulence—Chicken embryo infection model

The estimation of the virulence capacity of strain was performed by using the Chicken embryo as a model. The experiment was conducted for a few selected strains, where MRSA MTCC1430 served as a positive control and saline (0.7% NaCl) as a negative control. The experiment was carried out according to the protocol of Polakowska et al., [18] with few modifications, with the hatching eggs obtained from commercial hatcheries after ensuring that the feed did not contain any antibiotics. The chicken embryos were infected with test *S. aureus* strains on the seventh day, after the incubation. The mean survival rates were noted from the first to the seventh day after the infection i.e. from the eleventh day after incubation to seventeenth day through candling. On the seventeenth day, the liver was dissected from euthanized embryos. Histopathological studies were done to check the liver damage caused by the pathogen. The formalin-fixed liver tissue cross-sections were stained with H&E and were examined for fibrosis in the liver. The extent of liver tissue damage was confirmed and the damaged liver tissue was carefully examined and analyzed in comparison to control panels, using a manual approach.

### Statistical analyses

The data analysis was performed on R v3.4.2 and MedCalc. Differences among means were considered significant when *P*-value ≤ 0.05. Epidemiological analyses were performed with ‘Epi Info™’ (Version 7.2, Centre for Disease Control and Prevention). The odds ratios (OR), confidence intervals (CI), and *P* values were calculated by the χ2-test with Yates correction.

### Ethical statement.

The chicken-embryo experiment was performed at Vascular Biology Laboratory at AU-KBC Research Centre, Anna University, Chennai. The experimental protocol for the use of chicken embryos was submitted to the Institutional Biosafety and Ethical Committee (IBEC) of the AU-KBC Research Centre. The experiment was conducted with prior intimation to IBEC and IBEC declared that no formal approval was necessary to perform these experiments.

## Results

### Characterization and prevalence analysis of livestock-associated *S. aureus*

Initially the strains collected from local livestock were enumerated in the laboratory for the characterization of *S. aureus*. The strains were identified based on the morphological features and PCR characterization with Staph specific primers. After PCR confirmation, fifty-five isolates from the bovine-associated and thirty-six isolates from small ruminant’s origin confirmed as *S. aureus*. Among these isolates, twenty-eight isolates confirmed as MRSA based on the presence of the *mecA* gene in their genome. Phenotypic and molecular characterization confirms the isolated bacterial strains from the livestock were *S. aureus* strains. A multiplex PCR was performed assess the strain’s methicillin resistance at the molecular- level. In this set *SSCmec* and *tetM* primers used to confirm the presence of resistance genes in the isolates and further antibiogram results also indicate the resistance features of isolates towards commonly used antimicrobial drugs.

**Fig. 2:**
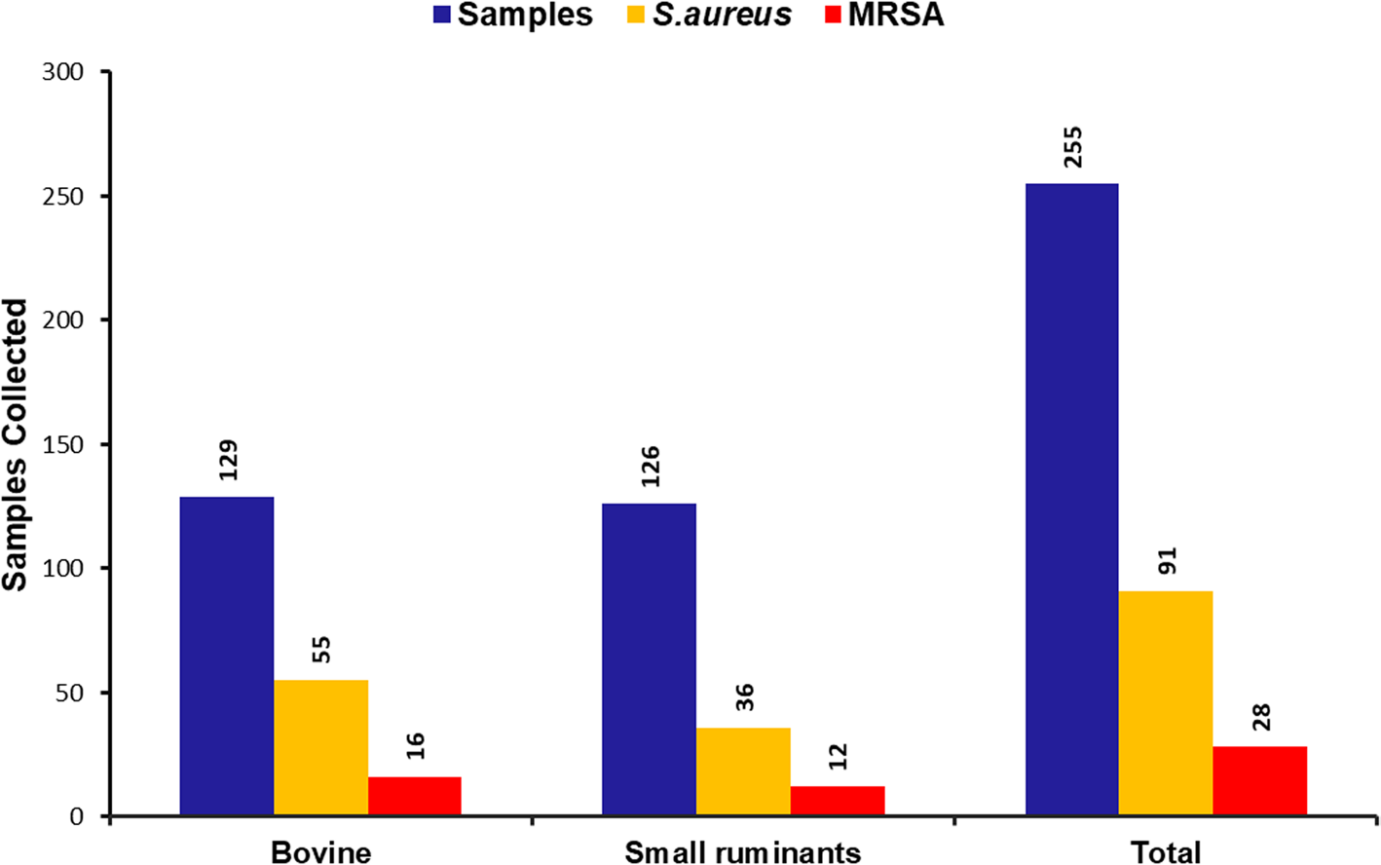
Prevalence analysis—Bar diagram representing the number of samples collected for prevalence analysis of *S. aureus* and MRSA in bovine and small ruminant’s samples

After molecular confirmation, the prevalence rate of *S. aureus* in the study population was analyzed. In this study, the prevalence rate in bovine-associated samples was 42.63% while it is low in the case of small ruminants-associated samples with 28.57% only. The bar diagram (Fig. 2) depicts the prevalence rate of LA*-SA* in the study population along with the MRSA prevalence rates. The chi-square test showed there is a significant difference in the prevalence of *S. aureus* between the two strains (χ^2^ = 5.49, *p*-value = 0.019). The overall prevalence of *S. aureus* found to 35.6% and MRSA 10.98% among the study samples.

The overall prevalence data presented in Table 1 with all calculated chi-square values (Yates Corrected χ2) and Odds Ratio (OR). Table 2 depicts the compared differences in the prevalence rates of *S. aureus* as well as MRSA with respect to the hosts. These results proved that there is a high probability of recovering *S. aureus* from livestock samples. But there is a high chance of recovering LA-MRSA from Small ruminant origin samples. However, there is no significant difference for isolating *S. aureus* as well as LA-MRSA strains from both the hosts in the study (Table 2). Further, the samples were categorized into urban- and rural- backgrounds, based on the area of collection to identify the influence of environment on the prevalence of *S. aureus* in the study population. The results revealed that urban environments have a high prevalence rate of *S. aureus* (Table 3).

**Table 1:** Statistics of the prevalence of *S. aureus* and MRSA isolates

Sample Details	OR	95% CI	χ2^^^	p-value
Total Samples (*n* = 255)	0.3079	0.2143 – 0.4423	40.658	0.0000
Bovine Associated (*n* = 129)	0.5524	0.3372 – 0.9049	5.023	0.0250
Small Ruminants Associated (*n* = 126)	0.163	0.0924 – 0.2763	44.587	0.0000
MRSA (Overall)	0.1975	0.1052 – 0.3702	25.406	1.23*10^–7^
MRSA (Bovine Associated)	0.1683	0.0739 – 0.3833	17.600	2.72*10^–6^
MRSA (Small Ruminants Associated)	0.250	0.0938 – 0.6613	6.722	0.00952

**Table 2:** Comparing frequency counts between two hosts for the prevalence of *S. aureus* and LA-MRSA

Sample Origin	Positive *(S. aureus*)	Negative	z for 95% CI	χ2	p-value
Bovine	55	74	1.96	5.493	0.0191
Small Ruminants	36	90			
MRSA (Bovine Origin)	16	39	1.96	0.184	0.668
MRSA (SR Origin)	12	24

**Table 3:** Statistics of the prevalence of *S. aureus* and MRSA isolates in rural and urban background environments

Sample Origin	Samples	Positive	OR	95% CI	χ2^^^	p-value
Rural	192	57	0.178	0.1151 – 0.2762	61.76	0.000
Urban	63	34	1.3746	0.6821 – 2.7698	0.5079	0.476
MRSA (Rural = 13)			0.0873	0.0364 – 0.0873	31.57	0.000
MRSA (Urban = 15)			0.6233	0.2393 – 1.6235	0.5294	0.466

Yates Corrected

### Antibiogram

**Fig. 3:**
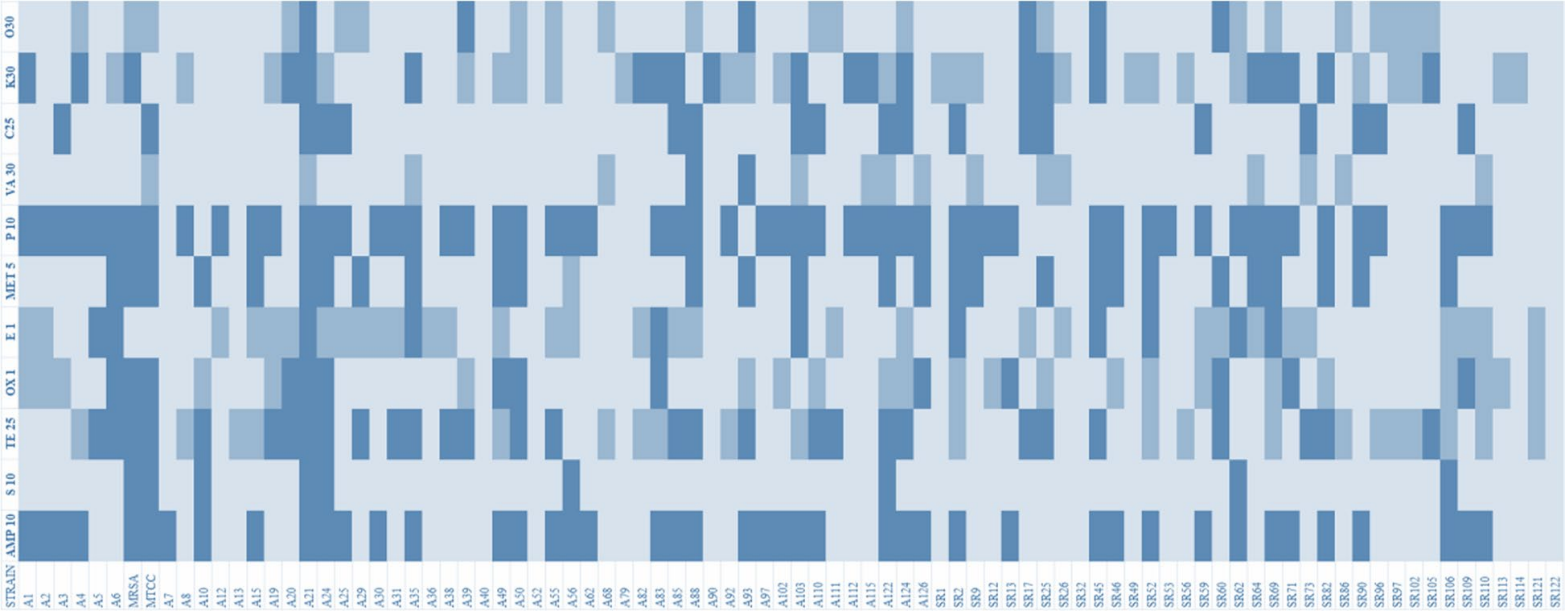
Heatmap of antibiotic resistance patterns—It describes the antibiotic resistance patterns among the livestock-associated isolates of this study. The dark blue cells represent resistant strains while the dark cyan and cyan cells represent intermediate and sensitive strains respectively

The antimicrobial susceptibility and resistant pattern results are straight forward to show the strain’s nature. In this study, the sensitivity of twelve different antibiotics covering major classes of antibiotics like beta-lactams, macrolides, etc. were assessed. The antibiotic-resistant patterns of various antibiotics used in this study have represented Table 4 with the number of antibiotics used and in the form of a heat map for better visualization (Fig. 3).

**Table 4:** Antimicrobial susceptibility profiles of LA*-SA* (*n* = 86)

Antibiotic Groups	Antibiotic	Conc	Resistant	Intermediate	Sensitive
β-lactams	Ampicillin (AMP)	10 µg	42(48.83%)	0(0.00%)	44(51.16%)
	Penicillin (P)	10 units	57(66.27%)	0(0.00%)	29(33.72%)
	Methicillin (MET)	5 µg	26(32.23%)	0(0.00%)	60(69.37%)
	Oxacillin (OX)	1 µg	12(13.95%)	23(26.74%)	41(47.67%)
Aminoglycosides	Kanamycin (K)	30 µg	21(24.41%)	26(32.23%)	39(45.34%)
	Streptomycin (S)	10 µg	7(8.13%)	0(0.00%)	79(91.86%)
Tetracyclines	Tetracycline (TE)	25 µg	28(32.55%)	21(24.41%)	37(43.02%)
	Oxytetracycline (O)	30 µg	6(6.97%)	19(20.93%)	61(70.93%)
Amphenicols	Chloramphenicol (C)	25 µg	19(20.93%)	0(0.00%)	68(79.07%)
Macrolide	Erythromycin (E)	15 µg	11(12.79%)	32(37.20%)	43(50%)
Glycopeptides	Vancomycin (VA)	30 µg	2(2.32%)	14(16.27%)	70(81.39%)

The isolated *S. aureus* strains were resistant to different classes of antibiotics. The study population is mostly sensitive to protein synthesis targeting drugs like Chloramphenicol, Streptomycin, and erythromycin, except tetracycline. But the study population is resistant to penicillin-like drugs or β-lactams. The results indicate the presence of MRSA and MDR-*SA* in the study population. The strains were showing different levels of resistance and sensitivity for methicillin. Methicillin resistance is defined as the strains of *S. aureus* that are resistant to the isoxazolyl penicillins, such as methicillin, oxacillin, and flucloxacillin [19]. The isolates were strongly susceptible to Streptomycin (91%), Vancomycin (81%), followed by Chloramphenicol (79%). Vancomycin-Resistant *Staphylococcus aureus* (VRSA) is a rare find in the study population. The methicillin resistance in the study population noted as 32.23% and also cumulative resistance of both methicillin and oxacillin resistance was found to be similar but with a meager increase in the resistance that is 32.56%. The production of the β-lactamase enzyme tested using an iodometric method using the starch-iodine indicator. The production of β-lactamase decolorizes the blue color developed after the addition of the starch-iodine indicator. It was noted that 57/88 strains produced the β-lactamase enzyme and in turn, these fifty-seven isolates became resistant to Ampicillin and Penicillin or Methicillin.

### Prevalence of Staphylococcal enterotoxins

Multiplex PCR, comprising six gene primers, was performed to find out the prevalence of SEs in the study population. The PCR amplifications were recorded on agarose gel electrograph for all the classical enterotoxin genes, except for enterotoxin D (*sed* – 278 bp), and for Toxic Shock Syndrome Toxin (TSST-1) encoding gene (*tst*—326 bp). Table 5 provides the details about the presence of toxin genes in the study population. Bovine-associated strains possess more toxin genes while small ruminants-associated strains lack most of the toxin genes.

**Table 5:** Prevalence of toxin genes in *S. aureus* isolates from different hosts

		No. of Positives	
**Gene**	**Product**	**Bovine (*n* = 55)**	**Small Ruminants (*n* = 36)**
*tst*	Toxic shock syndrome toxin 1	50.90 (28)	30.55 (11)
*sea*	Enterotoxin A	81.82 (45)	52.77 (19)
*seb*	Enterotoxin B	20.00 (11)	2.77 (1)
*sec*	Enterotoxin C	14.55 (8)	11.11 (4)
*sed*	Enterotoxin D	0 (0)	0 (0)
*see*	Enterotoxin E	52.72 (29)	33.34 (12)

TSST-1 encoding gene *tst,* along with enterotoxin a (*sea*) followed by enterotoxin e (*see*) was more prevalent in the study population while the other genes were scanty. Enterotoxin D encoding gene *sed* was completely absent in the study population. The association statistics are given in Table 5; it is evident, from these results that Enterotoxin A and TSST-1 genes were strongly associated, and enterotoxin E was moderately associated, with the study population isolates. Based on the epidemiological data of toxin genes in the study population, the host-based intra comparison was made to find the reservoir of toxigenic *S. aureus*. The chi-square based comparison showed that bovine-associated *S. aureus* (BA-SA) strains were more toxigenic (χ2 = 7.73, *p*-value = 0.0054) and can act as a reservoir for toxigenic *S. aureus*. The results strongly suggest that bovines colonize with toxigenic *S. aureus* strains.

### Biofilm formation capacity

**Fig. 4:**
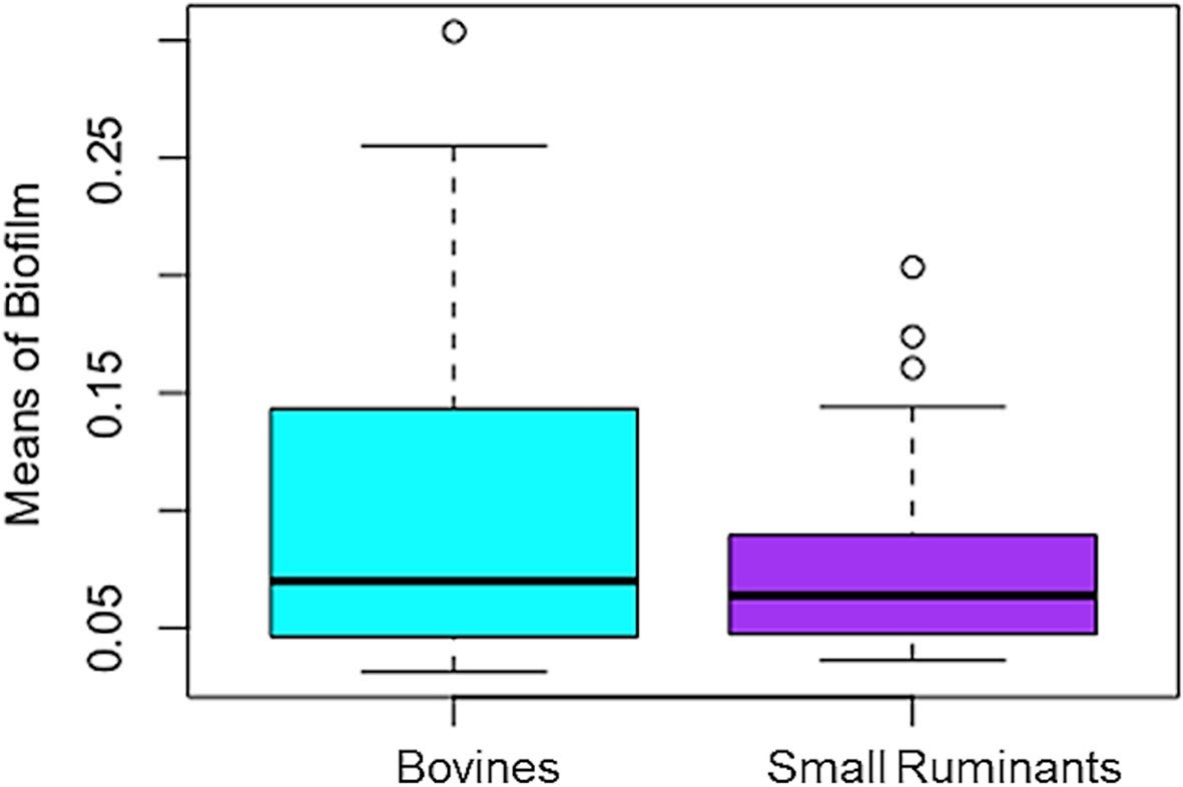
Box Plots of Biofilm means—The “R” generated boxplots of biofilm quantification analysis depict there was significant variation among bovine-associated and small ruminants-associated strains

Biofilm formation is a potential virulence factor in *S. aureus* isolated from clinical mastitis [20]. So, in order to have a comprehending command about the disease establishment by *S. aureus* strains from healthy animals, the biofilm formation assessment was carried out. The results showed that the strains were weak to the high capacity of biofilm formation. A parametric statistic test, Welch two-sample t-test was used to detect the variation of biofilm formation among the specificity. The Welch two-sample t-test was found to be statistically significant (Fig. 4, *t* = 2.0116, df = 80.4, and *p*-value = 0.0476). This result suggests that the BA-SA strains have a better capacity of biofilm formation than the small ruminants-associated strains.

**Fig. 5:**
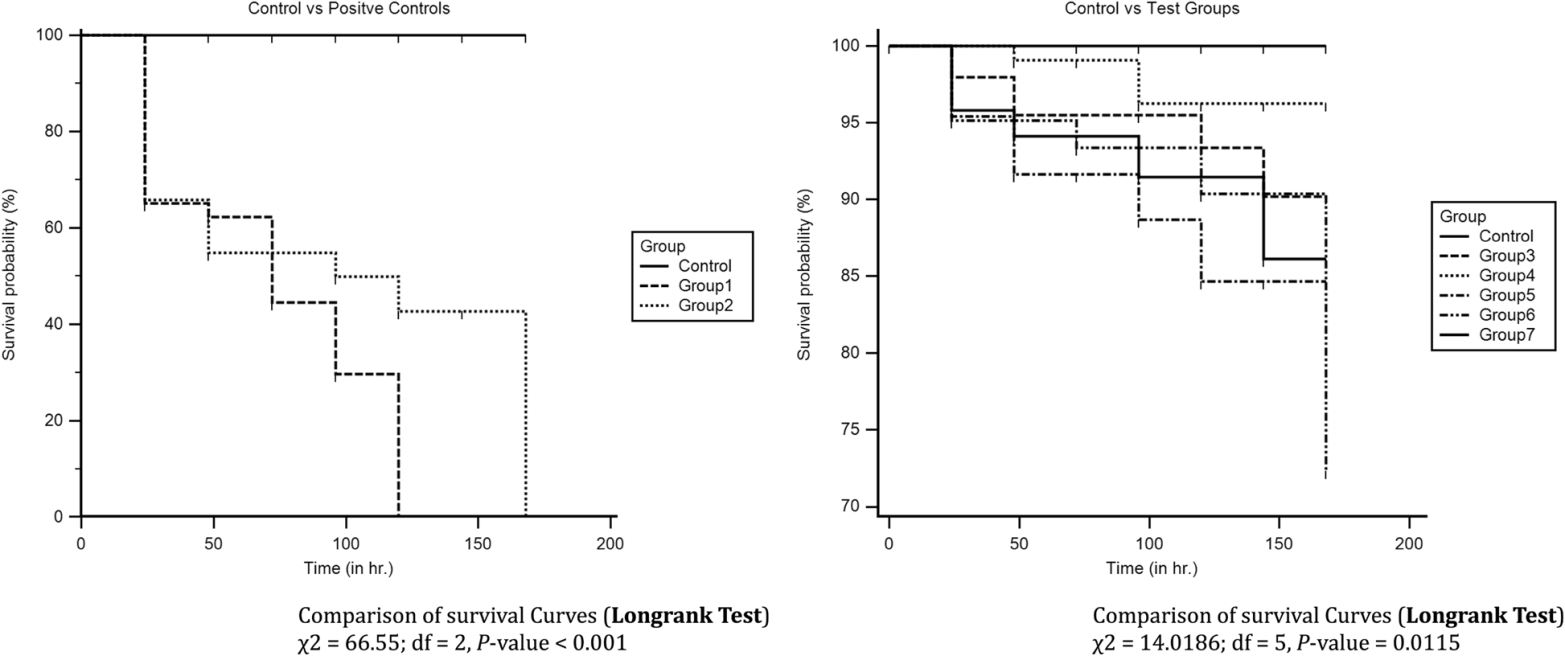
Survival Analysis—Kaplan–Meier curves representing the expected survival probability. The curves representing the survival rates of chicken embryos from day 1 to day 7 after challenging with *S. aureus*. Control: 0.7% NaCl, Group I – MRSA, Group II – Human-associated *S. aureus*, Group – III and IV – Small Ruminants-associated *S. aureus* (Sample IDs—SR32 and SR45 respectively); Group V – VII – BA-SA strains (Sample IDs—A21, A7, and A85 respectively)

On the basis of the antibiotic resistance, the biofilm formation capacity, and the toxicogenic capacity of strains, two strains from small ruminant-associated samples (with sample IDs – SR32 and SR42) and three strains from bovine-associated samples (with sample IDs – A21, A7, and A85) were selected for in vivo virulent assessment and for inter- and intra- comparisons. The survival analysis of embryos is depicted in Fig. 5.

**Fig. 6:**
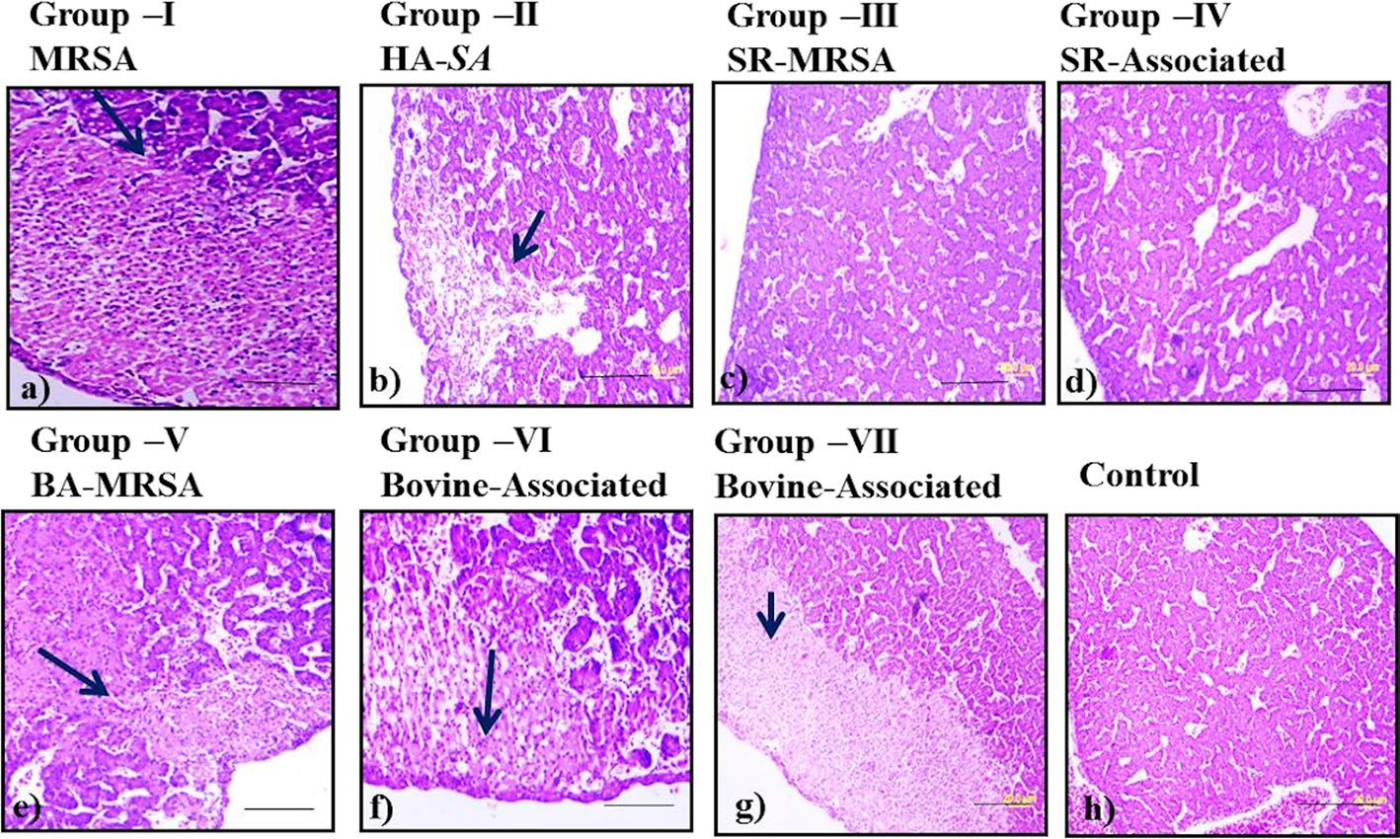
Histopathology photomicrographs—Histopathology analysis of liver-cross sections of chicken embryos showed liver fibrosis; fibrosis condition was shown with an arrow mark and scale bars for images = 20 μm. **a** Liver histology showed fibrosis in Group – I chicken embryo infected with MRSA MTCC1430 strain. **b** Fibrosis was observed in Liver cross-section of Group – II chicken embryo infected with Human-associated *S. aureus* strain. **c** & **d** Liver histology did not show fibrosis in Group – III & Group – IV chicken embryos infected with small ruminants- associated *S. aureus* strains SR32 and SR45, respectively. **e** Fibrosis was observed in Liver cross-section of Group – V chicken embryo infected with Bovine-associated methicillin-resistant *S. aureus* (BA-MRSA–A21) strain. **f** & **g** Liver histology did not show fibrosis in Group – VI & Group – VII chicken embryos infected with small ruminants- associated *S. aureus* strains A7 and A85 respectively, and **h** No fibrosis observed in liver cross-sections of chicken embryo control group (sterile 0.7% NaCl)

As expected, with respect to the control, fibrosis was observed in liver samples challenged with the pathogen but in the liver tissue cross-sections which were challenged with small ruminants-associated *S. aureus* strains, no fibrosis was observed. Clear fibrosis was observed in liver tissues challenged with MRSA, Human-associated *S. aureus*, and BA-*SA* (Fig. 6). The histopathological study supported the earlier biofilm formation and chicken embryo assays. The results highlighted the pathogenic potentiality and toxigenic nature of LA-*SA* isolates. The intra-comparison between bovine and small ruminant hosts suggested that bovine-associated strains were more pathogenic in nature.

## Discussion

The present study focused on the epidemiological and virulence characterization of Livestock-associated strains of this bacterium. Nasal-swabs were used as samples since it is the prescribed sampling method [21]. Molecular characterization of *S. aureus* performed with established protocols in accordance with previous studies. In this study, *Staphylococcus* specific *16S rRNA, nuc* and *sdrE* gene primers used to confirm the bacterial isolate as *S. aureus*. Duran et al., 2012 [22], developed multiplex PCR using *16S rDNA, mecA* and *femA* gene primers to characterize *S. aureus*. Meanwhile, the widely used gene primers were also used to characterize MRSA strain such as *nuc* and *mecA* in this study [11–13].

### Epidemiological and antibiogram analyses

The prevalence analysis suggests that bovine hosts could be potential reservoirs for *S. aureus* as well as LA-MRSA when compared with other small ruminants like sheep and goats. The majority of the prevailing reports focused mainly on the human origin and bovine mastitis associated with *S. aureus* [23, 24] but in recent times, the studies on epidemiological investigations of *S. aureus* from Livestock have increased. The prevalence analysis results are comparable to other previous studies. Mork et al., [21] reported that 16.0% of sheep and 58.2% of bovine nasal swab samples tested positive for the *S. aureus* strains. But a high prevalence rate of 43.24% for *S. aureus* strains was recorded from goat nasal swabs in Chongqing, China [25]**.**

The other similar studies also reported that Livestock can act as reservoirs for Livestock-Associated Methicillin Resistant *Staphylococcus aureus* (LA-MRSA). Basically, the swine populations are major reservoirs for *S. aureus* in comparison with other farm animals and livestock. The prevalence of LA-MRSA-positive pig farms in Poland has increased considerably since 2008. Nasal carriage of LA*-SA* by pig farmers at Poland estimated at 13.2% [26]. But a high percentage of LA-MRSA in pigs (65%) was reported in the. Many of the studies suggest that pigs can act as reservoirs for LA-*SA* but the study by Verstappen et al., [27] suggests that Bovines can also act as reservoirs for *S. aureus*. Along with the livestock, increased incidence of LA-*SA* from 2016 in humans was also observed in Kuwait [28].

Uncontrolled and over usage of antibiotics resulted in antibiotic resistance in *S. aureus*. The study population is mostly sensitive to protein-synthesis targeting drugs like Chloramphenicol, Streptomycin, and erythromycin but resistant to β-lactams. Penicillin class antibiotics are highly used in veterinary medicine and as a feed additive in India (Centre for Science and Environment – CSE, 2014). Considerable rates of antibiotic resistance patterns were observed in this study. Overall, a higher rate of methicillin resistance (30.78%) was observed as compared to previous reports reporting a range only 5 to 29%, in Indian cattle and buffalo isolates [29–31]. A similar range of resistance- rates were recorded for LA-MRSA in cattle in other countries, i.e. 19.8% in Belgium, 34% in cattle of Faisalabad of Pakistan and 61% in Italy [32–34].

It is mandatory to assess the virulence capacity of an opportunistic pathogen. The virulence capacity of a strain of such a pathogen can be determined with several molecular, in vitro and in vivo studies. *S. aureus is* considered to be one of the bacteria that form a strong biofilm which in turn helps *S. aureus* to gain resistance against antimicrobials [35]. Biofilm formation also is regarded as a potential virulence factor in *S. aureus* by helping the bacteria with the adherence and establishing the infection. Hence, quantifying the biofilm gives an overview of the strains' pathogenicity.

Quantification of biofilm revealed that the bovine-associated strains appear more successful at forming biofilm than the small ruminants-associated strains. The reason behind this is not yet established. Lee et al., [36] , assessed the biofilm-producing ability of *S. aureus* isolates from Brazilian dairy farms. That study reported the prevalence rate of *S. aureus* as 45% in dairy farms, and the biofilm-forming capacity of the isolated strains was found comparatively lesser than the MRSA-type strains in the laboratory. In general, MRSA strains form biofilm at a higher rate as reported by Cha et al., (2013) [37].

Staphylococcal super-antigens (SAgs), such as TSST-1, are the main cause of toxic shock syndrome. The prevalence of the *tst* gene in clinical samples of Wegener's Granulomatosis of Netherlands was reported to be around 24% [38]. In an analysis of virulence genes among MRSA strains, a study conducted at Iran found that the MRSA strains harbor low frequency (2.5%) of *tst* genes [39]. When it comes to livestock aspect not many studies are available in order to compare the prevalence of toxin genes. In Italy, a study specifically on ST1 of LA-*SA* lineage observed that *sea* gene was found in 9 out of 20 strains from cattle, herds, and humans [40]. Another study from Northern Brazil reported that 65.78% of the *S. aureus* isolates carried toxin genes, including *seb, sec, sed,* and *tst* [41]. The presence of SEs in the strain's genome indicates the presence of pathogenic strains, in the study population. This study reported that bovine-associated strains are possessed more virulent genes than small ruminants-associated strains. The reason behind this is not yet established, but the most plausible reason may be the mastitis disease which is very common in bovines. In India, the prevalence rate of bovine mastitis is found to be in the range of 34.56% to 51.43% [42]. Another explanation that can be made from the field observations is that cows are domestic animals with frequent human contact, while small ruminants mostly live in herds. The chance for a human-to-animal transmission of *S. aureus* cannot be ruled out.

Chicken embryo assays are employed to investigate the pathogenesis of *S. aureus* from earlier times for studying bacterial interference between the strains of *S. aureus*, in developing an experimental model of bacterial chondronecrosis, and to study internalization of *S. aureus* in osteomyelitis [43, 44]. With reference to findings of Polakowska et al.,, the colonization was found to be less in small ruminants-associated strains and moderate in bovine-associated strains. The moderate colonization and less pathogenic potentiality presumably are due to lack of the adherence genes that are essential for the establishment of the disease or colonization of the host tissue or the host immune system. These results are in accordance with the post-infection survival rate of embryos reported by Polakowska et al., The survival rate varies from 60–80% in those groups that were infected with bovine-associated strains. As expected, MRSA-type strain (MTCC1430) exhibited more virulence; however, at the end of the experiment, the survival rate was 0%. Since, the MRSA-type strain known to possess maximum known virulence factors, the survival rate of embryos was expected to be 0% at the end of the experiment.

With these analyses, it was found that LA-*SA* strains contain toxic genes and are pathogenic in nature especially bovine-associated strains. The small ruminants-associated strains were comparatively less toxigenic and pathogenic in nature; however, the exact reason for this type of behavior is yet to be established. The bovines were colonized with toxigenic *S. aureus* which could act as reservoirs. This study is in agreement with Springer et al., (2009) [45], which suggested that MRSA could be a novel zoonotic agent and suggests that LA-*SA* strains could be a novel zoonotic agent. Another study as well suggested that ST1 lineage of LA-*SA* strains isolated from farm animals could be a potential threat to public health [40].

## Conclusion

This study was designed to investigate the incidence of *S. aureus* in economically important livestock animals. The study results indicate a prevalence rate of *S. aureus* was high in bovine origin samples than small ruminants. There is no significant difference was observed in the incidence of MRSA among bovines and small ruminants. The presence of MRSA and MDR-SA in domestic Livestock animals could be a potential threat to animal and public health. This study suggests bovines could act as reservoirs for *S. aureus*.

In order to predict the virulence capacity of strains, molecular and in vivo experiments were carried out. The prevalence analysis of classical enterotoxin genes in the study population also suggested that bovine-associated strains were highly toxigenic in nature and recorded high prevalence rates. Further, the in vivo assessment of the virulence capacity of *S. aureus* strains was carried out in the chicken embryo model since the chicken embryos were sterile and suitable model organism to study the pathogenesis of bacterial pathogens. All the experimental data suggested that BA-SA strains could be pathogenic in nature and bovines shall act as reservoirs for pathogenic and drug-resistant *S. aureus* strains, even though low pathogenicity recorded for small ruminant-associated isolates cannot be ignored during the course of evolution.

Further studies with more samples are needed to estimate the more realistic prevalence of *S. aureus* and MRSA in the study sites. This study shall be extended to characterize the pathogenic potential of LA-*SA* isolates from the study population and compared with the host specificity.

### Supplementary Information

**Additional file 1. **Primers used in multiplex PCR.

## Data Availability

Data is available under request.

## References

[1] Weese JS (2010). Methicillin-resistant Staphylococcus aureus in animals. ILAR J.

[2] Menzies PI, Ramanoon SZ (2001). Mastitis of sheep and goats. Vet Clin North Am Food Anim Pract.

[3] Bradley AJ (2002). Bovine mastitis: an evolving disease. Vet J.

[4] McNamee PT, McCullagh JJ, Rodgers JD, Thorp BH, Ball HJ, Connor TJ, McConaghy D, Smyth JA (1999). Development of an experimental model of bacterial chondronecrosis with osteomyelitis in broilers following exposure to Staphylococcus aureus by aerosol, and inoculation with chicken anemia and infectious bursal disease viruses. Avian Pathol.

[5] Viana D, Selva L, Segura P, Penades JR, Corpa JM (2007). Genotypic characterization of Staphylococcus aureus strains isolated from rabbit lesions. Vet Microbiol.

[6] Robinson TP, Bu DP, Carrique-Mas J, Fèvre EM, Gilbert M, Grace D, Hay SI, Jiwakanon J, Kakkar M, Kariuki S, et al. Antibiotic resistance is the quintessential One Health issue. Trans R Soc Trop Med Hyg. 2016;110:377–380. 10.1093/2Ftrstmh/2Ftrw048 10.1093/trstmh/trw048 PMC4975175 27475987

[7] McEwen SA, Fedorka-Cray PJ (2002). Antimicrobial use and resistance in animals. Clin Infect Dis.

[8] Butaye P, Argudín MA, Smith TC (2016). Livestock-associated MRSA and its current evolution. Curr Clin Microbiol Rep.

[9] Van Duin D, Paterson DL (2016). Multidrug-resistant bacteria in the community: trends and lessons learned. Infect Dis Clin.

[10] Schaumburg F, Mugisha L, Kappeller P, Fichtel C, Köck R, Köndgen S, Becker K, Boesch C, Peters G, Leendertz F (2013). Evaluation of non-invasive biological samples to monitor Staphylococcus aureus colonization in great apes and lemurs. PLoS One..

[11] Molla B, Byrne M, Abley M, Mathews J (2012). Epidemiology and Genotypic characterization of MRSA strains of porcine origin. J Clin Microbiol.

[12] Frana TS, Beahm AR, Hason BM, Kinyon JM, layman LL, Karriker LA, Ramirez A, Smith TC. Isolation Characterization of methicillin resistant Staphylococcus aureus isolated from pork farms and Visiting Veterinary Students. PLoS One. 2013;8:e53738. 10.1371/journal.pone.0053738 10.1371/journal.pone.0053738 PMC3536740 23301102

[13] Rahimi H, Saei HD, Ahmadi M. Nasal carriage of Staphylococcus aureus: Frequency and Antibiotic resistance in healthy ruminats. Jundisphur J Microbiol. 2015;8:e22413. 10.5812/2Fjjm.22413 10.5812/jjm.22413 PMC4640094 26568802

[14] Bauer AW, Kirby WM, Sherris JC, Turck M (1966). Antibiotic susceptibility testing by a standardized single disk method. Am J Clin Pathol.

[15] Rota C, Yangüela J, Blanco D, Carraminana JJ, Arino A, Herrera A (1996). High prevalence of multiple resistance to antibiotics in 144 Listeria isolates from Spanish dairy and meat products. J Food Prot.

[16] Isenberg HD (2004). Clinical Microbiology Procedure Handbook.

[17] O’Toole GA (2011). Microtiter dish biofilm formation assay. J Vis Exp.

[18] Polakowska K, Lis MW, Helbin WM, Dubin G, Dubin A, Niedziolka JW, Miedzobrodzki J, Wladyka B (2012). The virulence of Staphylococcus aureus correlates with strain genotype in a chicken embryo model but not a nematode model. Microb Infect.

[19] Loomba PS, Taneja J, Mishra B (2010). Methicillin and Vancomycin Resistant S. aureus in Hospitalized Patients. J Glob Infect Dis.

[20] Hensen SM, Pavičić MJAMP, Lohuis JACM, de Hoog JAM, Poutrel B (2000). Location of Staphylococcus aureus within the experimentally infected bovine udder and the expression of capsular polysaccharide type 5 in-situ. J Dairy Sci.

[21] Mørk T, Kvitle B, Mathisen T, Jørgensen HJ (2010). Bacteriological and molecular investigations of Staphylococcus aureus in dairy goats. Vet Microbiol.

[22] Duran N, Ozer B, Duran GG, Onlen Y, Demir C (2012). Antibiotic resistance genes & susceptibility patterns in Staphylococci. Indian J Med Res.

[23] Dubey D, Rath S, Sahu MC, Pattnaik L, Debata NK, Padhy RN. Surveillance of infection status of drug resistant Staphylococcus aureus in an Indian teaching hospital. Asian Pac J Trop Dis. 2013;3:133–142. 10.1016/2FS2222-1808(13)60057-2

[24] Gharsa H, Slama KB, Gomez-Sanz E, Lozano C, Zarazaga M, Messadi L, Boudabous A, Torres C (2015). Molecular characterization of Staphylococcus aureus from nasal samples of healthy farm animals and pets in Tunisia. Vector Borne Zoonotic Dis.

[25] Zhou Z, Zhang M, Li H, Yang H, Li X, Song X, Wang Z (2017). Prevalence and molecular characterization of Staphylococcus aureus isolated from goats in Chongqing. China BMC Vet Res.

[26] Mroczkowska A, Żmudzki J, Marszałek N, Orczykowska-Kotyna M, Komorowska I, Nowak A, Grzesiak A, Czyżewska-Dors E, Dors A, Pejsak Z (2017). Livestock-associated Staphylococcus aureus on Polish pig farms. PLoS One.

[27] Verstappen KM, Willems E, Fluit AC, Duim B, Martens M, Wagenaar JA (2017). Staphylococcus aureus nasal colonization Differs among Pig lineages and is associated with the Presence of Other staphylococcal species. Front Vet Sci.

[28] Boswihi SS, Udo EE, Mathew B, Noronha B, Verghese T, Tappa SB (2020). Livestock-Associated Methicillin-Resistant Staphylococcus aureus in Patients Admitted to Kuwait Hospitals in 2016–2017. Front Microbiol.

[29] Kumar R, Yadav BR, Anand SK, Singh RS (2011). Molecular surveillance of putative virulence factors and antibiotic resistance in Staphylococcus aureus isolates recovered from intra-mammary infections of river buffaloes. Microb Pathog.

[30] Prashanth K, Rao KR, Reddy PV, Saranathan R, Makki AR. Genotypic characterization of Staphylococcus aureus obtained from humans and bovine mastitis cases in India. J Glob Infect Dis. 2011;3:115–122. 10.4103/2F0974-777X.81686 10.4103/0974-777X.81686 PMC3125022 21731296

[31] Rajkhowa S, Sarma DK, Pegu SR (2016). SCC mec typing and antimicrobial resistance of methicillin-resistant Staphylococcus aureus (MRSA) from pigs of Northeast India. Vet Res Commun.

[32] Antoci E, Pinzone MR, Nunnari G, Stefani S, Cacopardo B (2013). Prevalence and molecular characteristics of methicillin-resistant Staphylococcus aureus (MRSA) among subjects working on bovine dairy farms. Infez Med.

[33] Nemeghaire S, Argudín MA, Haesebrouck F, Butaye P (2014). Epidemiology and molecular characterization of methicillin-resistant Staphylococcus aureus nasal carriage isolates from bovines. BMC Vet Res.

[34] Aqib AI, Ijaz M, Anjum AA, Malik MA, Mehmood K, Farooqi SH, Hussain K (2017). Antibiotic susceptibilities and prevalence of Methicillin Resistant Staphylococcus aureus (MRSA) isolated from bovine milk in Pakistan. Acta Trop.

[35] Gefen O, Balaban NQ (2009). The importance of being persistent: heterogeneity of bacterial populations under antibiotic stress. FEMS Microbiol Rev.

[36] Lee SHI, Mangolin BLC, Gonçalves JL, Neeff DV, Silva MP, Cruz AG, Oliveira CAF (2014). Biofilm producing ability of Staphylococcus aureus isolates from Brazilian dairy farms. J Dairy Sci.

[37] Cha JO, Yoo JI, Yoo JS, Chung HS, Park SH, Kim HS, Lee YS, Chung GT. Investigation of biofilm formation and its association with the molecular and clinical characteristics of methicillin-resistant Staphylococcus aureus. Osong Public Health Res Perspect. 2013;4:225–232. 10.1016/2Fj.phrp.2013.09.001 10.1016/j.phrp.2013.09.001 PMC3845227 24298437

[38] Deurenberg RH, Nieuwenhuis RF, Driessen C, London N, Stassen FR, Van Tiel FH, Stobberingh EE, Vink C (2005). The prevalence of the Staphylococcus aureus tst gene among community-and hospital-acquired strains and isolates from Wegener's Granulomatosis patients. FEMS Microbiol Lett.

[39] Alfatemi SM, Motamedifar M, Hadi N, Saraie HS (2014) Analysis of virulence genes among methicillin resistant Staphylococcus aureus (MRSA) strains. Jundishapur J Microbiol 7:e10741. 10.5812/jjm.10741 10.5812/jjm.10741 PMC4217665 25371805

[40] Alba P, Feltrin F, Cordaro G, Porrero MC, Kraushaar B, Argudín MA, Nykäsenoja S, Monaco M, Stegger M, Aarestrup FM, et al (2015) Livestock-associated methicillin resistant and methicillin susceptible Staphylococcus aureus sequence type (CC) 1 in European farmed animals: high genetic relatedness of isolates from Italian cattle herds and humans. PLoS One 10: e0137143. 10.1371/journal.pone.0137143 10.1371/journal.pone.0137143 PMC4556339 26322785

[41] Costa FN, Belo NO, Costa EA, Andrade GI, Pereira LS, Carvalho IA, Santos RL (2018). Frequency of enterotoxins, toxic shock syndrome toxin-1, and biofilm formation genes in Staphylococcus aureus isolates from cows with mastitis in the Northeast of Brazil. Trop Anim Health Pro.

[42] Patel JV, Bhingaradia BV, Patel BB, Patel SB, Patel PB, Vahora SP (2012). Study on Prevalence of Mastitis and Antibiotic Sensitivity of Bacterial Isolates Recovered from Crossbred Cows of Anand District of Gujarat. Indian J Dairy Sci.

[43] McNamee PT, Smyth JA (2000). Bacterial chondronecrosis with osteomyelitis ('femoral head necrosis') of broiler chickens: a review. Avian Pathol.

[44] Reilly SS, Hudson MC, Kellam JF, Ramp WK (2002). In vivo internalization of Staphylococcus aureus by embryonic chick osteoblasts. Bone.

[45] Springer B, Orendi U, Much P, Höger G, Ruppitsch W, Krziwanek K, Metz-Gercek S, Mittermayer H (2009). Methicillin-resistant Staphylococcus aureus: a new zoonotic agent?. Wien Klin Wochenschr.

