# Supplementary material for: Epidemiology and zoonotic potential of Livestock-associated Staphylococcus aureus isolated at Tamil Nadu, India
Source: BMC Microbiol. 2023 Nov 4;23:326. doi: 10.1186/s12866-023-03024-3 (PMC10625228; doi:10.1186/s12866-023-03024-3)
Supplement: Supplementary file 1 — Additional file 1. Primers used in multiplex PCR. [file 12866_2023_3024_MOESM1_ESM.docx]

**β-Lactamase production**

Since bacteria resistant to Penicillin class of antibiotics produce β-lactamase which in turn helps to support the data obtained from plate-based assays. For detecting the β-Lactamase iodometric method was used as per the American Society for Microbiology (ASM) protocol (Isenberg, 2004). In brief, *S. aureus* isolates were inoculated into freshly prepared sterile Luria-Bertani broth and incubated at 37°C for 18 – 24 hrs. A 250 μl of culture medium transferred to 1.5 ml centrifuge tube followed by the addition of 0.2 ml of 5% soluble starch and 20 μl of freshly prepared 5% penicillin G. After the components were mixed thoroughly, 30 μl of I_2_ (0.1 N) - KI (0.8 N) was added. After the addition of the Iodine solution, the sample turns to dark blue immediately. The discoloration of the sample occurs subsequently if there is the production of β-Lactamase.

**Primers used in Multiplex PCR**

| **Primer ID** | **Sequence** | **Product** | **Amplicon length** | **Reference** |
| --- | --- | --- | --- | --- |
| Staph-756 | **Forward Primer**  5’-AACTCTGTTATTAGGGAAGAACA-3’ | Staphylococcal 16s *rRNA* | 756 bp | McClure et al., 2006 |
|  | **Reverse Primer**  5’-CCACCTTCCTCCGGTTTGTCA CC-3’ |  |  |  |
| *tetM* | **Forward Primer**  5’-AGTGGAGCGATTACAGAA-3’ | *tetM* | 258 bp | McCarthy et al., 2014 |
|  | **Reverse Primer**  5’-CATATGTCCTGGCGTGTCTA-3’ |  |  |  |
| *femA* | **Forward Primer**  5’-CGATCCATATTTACCATATCA-3’ | *femA* | 535 bp | Al-Talib et al., 2009 |
|  | **Reverse Primer**  5’-ATCACGCTCTTCGTTTAGTT-3’ |  |  |  |
